# Nurse educators and student nurses’ perspectives on ways to improve implementation of simulation-based education in Lesotho

**DOI:** 10.4102/curationis.v45i1.2260

**Published:** 2022-05-30

**Authors:** Pule S. Moabi, Ntombifiakile G. Mtshali

**Affiliations:** 1 Department of Nursing, Scott College of Nursing, Morija, Lesotho; 2 Department of Nursing, University of KwaZulu-Natal, Durban, South Africa

**Keywords:** simulation, students, nurse educator, clinical supervisor, nursing education institutions, preceptor, simulation facilitator, resources

## Abstract

**Background:**

In order to ensure an effective health system, there is a need to recruit, train and deploy a competent nursing workforce. A competent workforce can be made possible by integrating simulation into the curriculum. Implementation of simulation-based education in Lesotho is facing a number of challenges as the country has limited resources.

**Objectives:**

This study aimed to describe nurse educators and students’ perspectives on ways to improve implementation of simulation-based education in Lesotho.

**Method:**

A qualitative study was conducted. A total of 24 students, 24 nurse educators and 4 principals who were purposely selected participated in the study. Focus group discussions as one of the data collection methods were used to collect data from the nurse educators and students whilst in-depth, unstructured individual interviews were used with the principals. Data were analysed following the Corbin and Strauss grounded theory approach where similar codes were categorised together as part of open coding, and axial coding was conducted by refining the codes and organising them into categories and subcategories.

**Results:**

Two categories emerged from the areas where improvement is required: resources to support simulation. Resources emerged as playing a major role in ensuring quality simulation. The teaching and learning process emerged as collaborative in nature with all key players ensuring that they meet their responsibilities in order to ensure effective simulation-based learning.

**Conclusion:**

The study revealed that there are limited numbers of simulation facilitators and this hinders effective implementation of simulation. Students are concerned about the comments of educators during simulation, as some of the comments are belittling.

## Introduction

Effective health systems are essential to the health of the population, and in order to ensure an effective health system, there is a need to recruit, train and deploy a competent nursing workforce. Improving the quality of nursing education may lead to the strengthening of health systems (World Health Organisation [WHO] 2011:4). In order to ensure that competent students graduate from the Nursing Education Institutions (NEIs), the WHO (2013:37) recommends that simulation needs to be adopted. High-fidelity simulation, as one of the clinical teaching methodologies, can be used in institutions with adequate resources whilst the low-fidelity simulation can be used in resource-limited settings (WHO 2013:36). There are various debates on the number of hours that can be allocated to simulation, as Dobrowolska et al. (2015:8) contended that in England, the number of hours allocated for simulation is 300, which accounts to 13% of the total curriculum hours, whilst in Slovenia 10% – 15% of the total clinical time of 2300 h can be spent in the simulation laboratories practising skills. In Lesotho, a newly adopted competency-based curriculum in the diploma in nursing programme shows that work integrated learning (WIL) at the first-year and second-year level is allocated 16 h per week, whilst in the third year, WIL is allocated 24 h per week (Botma et al. 2012:35). These authors further stated that within those allocated hours, simulation must form part of WIL even though no guidance is provided on how much time is needed for simulation (Botma et al. 2012:35). When simulation-based education (SBE) is integrated into the competency-based curriculum, graduates will be able to provide competent, culturally sensitive and evidence-based nursing services (WHO 2011:6).

Martins et al. (2018:8) defined simulation as a teaching strategy where various educational methods and types of equipment are used to provide a simulated experience to the students assisting them to progress from novice to expert levels. This is supported by Jeffries, Rodgers and Adamson (2015:97), who explained that in simulation, the environment must be experiential, interactive, collaborative and learner-centred, and the environment must allow students to practice skills and decision-making skills. Learning in a simulated environment has various advantages to the nursing student, the patients and the clinical areas as it assists nursing students to learn and apply clinical decision-making skills and critical reasoning in a safe environment, where a patient’s life is not put at risk (Larue, Pepin & Allard 2015:134; Liaw et al. 2014:1050).

There are number of learning theories that support use of SBE in nursing (Belanger 2011:51; Mukhalati & Taylor 2019:7). These include adult learning theory, which describes ways in which adults assimilate knowledge, skills and attitudes and states that adults need to be actively involved in their learning (Merriam & Bierema 2014:50). Active involvement of the students according to Guinez-Molinos et al. (2017:197) is one of the key components to ensure successful implementation of SBE. Active involvement of students in simulation leads to reduced anxiety and increased confidence and enhances therapeutic communication (Carpenter, Hirthler & King 2018:238; Palmer & Ham 2017:282). Students should be given an opportunity to collaboratively design and perform activities in simulated environments in order to promote active involvement (Guinez-Molinos et al. 2017:196).

One of the theories supporting SBE explains that individuals construct new knowledge through interaction between their previous skills and knowledge, which is called constructivist learning, according to Mukhalati and Taylor (2019:7). Based on the learner’s environment, knowledge is actively constructed and the environment needs to be structured in a manner that supports learning (Belanger 2011:51). According to Baptista, Pereira and Martins (2016:13), educators must be innovative and ensure realism in the simulation laboratory to ensure constructivism. A study conducted by Woodruff, O’Neil and Walton-Moss (2017:349) found out that realism in simulation was an issue to other students, as they stated that the simulators were not close to reality. This implies that students felt that the simulators do not respond like real patients as they have restricted responses.

One of the theories that supports SBE is experiential learning where adult learners learn through hands-on activities (Belanger 2011:51; Merriam & Bierema 2014:41). During these hands-on activities, they need supervision from experienced facilitators. A study conducted by Krishnan, Keloth and Ubedulla (2017:85) showed that simulation needs dedicated educators or clinical instructors, and the ideal ratio of the clinical instructor to the student must be 1:3. The ratio in Lesotho is 1:20, and this poses a challenge as students are inadequately supervised (Nursing Education Partnership Initiative [NEPI] 2012:6). In addition, financial resources need to be considered when planning and conducting simulation, as manikins (especially the high-fidelity ones) are very expensive to buy and maintain (Krishnan et al. 2017:86). Within the simulation laboratories, real medical equipment is used for simulation purposes, but some nursing schools cannot afford to buy such (Martins et al. 2018:15)

According to Krishnan et al. (2017:86) and Larue et al. (2015:134), SBE especially when using the task trainers such as intramuscular injection trainer requires use of real medical equipment and supplies. This implies that institutions need to procure the medical equipment and supplies in order to ensure realism during simulation. The manikins need to be maintained and serviced regularly, faculty needs to be continuously capacitated on simulation and there is need for ongoing administrative and technical support to simulation facilitators. All these actions require financial resources and some institutions may not have adequate financial resources to support SBE. Furthermore, nursing regulatory bodies or the curriculum needs to provide guidance on the number of hours a student needs to complete in simulation (South Africa Nursing Council circular 2020).

In Lesotho, simulation was first introduced as a teaching methodology in 2012 (NEPI 2012:1). With the current implementation of SBE in Lesotho, NEIs use improvised equipment and supplies during simulation, malfunctioning high-fidelity manikins are not fixed timeously, leading to poor simulation, and simulation laboratories are manned by educators who also conduct demonstrations and do students’ follow-ups in the clinical areas (Munangatire & Naidoo 2017:45). In Lesotho, there is no formal guidance on how many clinical hours a nursing student needs to spend in the simulation laboratory in the newly developed curriculum (Botma et al. 2012:35), there are no guidelines from the Lesotho Nursing Council on the number of hours a student needs to spend on simulation and students are only assessed on how to carry out ‘procedures’ whilst critical thinking, decision-making, problem solving and teamwork are ignored (NEPI 2012:6). The study aimed at describing nurse educators and students’ perspectives on ways to improve implementation of SBE in Lesotho. Grounded theory approach assisted the researchers to explore the little-known phenomenon, that being ways of improving implementation of SBE in Lesotho, which is a resource-constrained setting (Corbin & Strauss 2015:92; Tie, Birks & Francis 2019:2). The study was guided by the following research question: ‘what are the perceptions of nurse educators and student nurses on ways to improve implementation of SBE in Lesotho?’

## Methods

### Research design

A qualitative grounded theory approach was used. Grounded theory is defined as a structured and flexible qualitative research approach that enables the researcher to study a phenomenon and develop new theory based on the collected data (Tie et al. 2019:2). Grounded theory approach is considered the best approach to address the gap, because ways of improving SBE implementation are little-known phenomena in Lesotho. The end product of the study is to construct a theory from the results, which will be published later in a separate article (Corbin & Strauss 2015:105; Tie et al. 2019:1).

### Research setting

The study was conducted in four private NEIs in Lesotho. These NEIs utilise a competency-based curriculum which is learner-centred. The institutions run similar programmes, a 3-year nursing diploma and a 1-year midwifery diploma, using SBE as one of their clinical teaching methodologies. Public NEIs were not included because they do not use competency-based curricula and some offer qualifications higher than diploma.

### Population and sampling

In this study, the population comprised eight second-year, eight third-year and eight midwifery students, 24 nurse educators and four principal nurse educators who were purposely selected because they had been exposed to SBE and high-fidelity manikins (HFMs). For recruitment of the participants, within each NEI there was a focal person who provided emails of the nurse educators and the principals. Students were recruited via Whatsapp where the focal persons added the researchers to the class Whatsapp groups. Within those platforms (emails and Whatsapp groups), the researchers wrote messages informing potential participants about the purpose of the study, issues relating to confidentiality and how data were to be collected and appointments were secured.

### Pilot study

A pilot study was performed by conducting two focus group discussions (FGD), one for nurse educators and one for students. One in-depth, face-to-face, unstructured individual interview was also conducted with the principal nurse educator. The participants during the pilot stage provided relevant responses, and no modifications were carried out on the data collection instruments; their results formed part of the main study. This is in line with what Gray, Grove and Sutherland (2017:54) recommended, as during the pilot study, if there are no modifications needed to the data collection instruments, the data obtained from the pilot study can be included in the main study. These pilot interviews were performed to test feasibility of the study and to assess the appropriateness and quality of the data collection instruments (Polit & Beck 2017:195).

### Data collection

Focus group discussions (FGDs) with the aid of an interview guide were used to collect data from nurse educators and students. Four FGDs were conducted for both nurse educators and students, culminating in a total of eight FGDs, each FGD comprising of six participants. During the FGD, the participants were seated in a semicircle in order to maintain face-to-face contact. The topic of the day was explained, participants were given codes, an introductory question was asked and the central question allowed the discussions to flow. Each focus group discussion was led by a moderator (first author P.S.M.) and the FGD lasted between 45 min and 90 min. To ensure that all participants participated in the FGD, the moderator encouraged inputs from the less vocal participants, directed questions to the quiet participants and maintained eye contact with the nonvocal members of the FGD to signal them that the moderator was interested in hearing from them (Andrew & Halcomb 2009:73). For the principal nurse educators, in-depth, face-to-face, unstructured individual interviews and field notes were used to collect data; the interviews were recorded with a voice recorder. The interviews were conducted from 25 August 2020 to 05 January 2021. The interviews were guided by the following central question: ‘how can simulated learning be improved?’ In order to ensure that the central question was adequately addressed during the interviews, the researchers used facilitative communication skills, which included reflecting the content, questioning, probing and paraphrasing, as outlined by Moabi and Mavundla (2018:3). As the interviews were conducted during the coronavirus disease 2019 (COVID-19) pandemic, a distance of 1 m was maintained, all participants had face masks on and the windows were opened. Before and after analysis, data were kept under lock and key to ensure that only the researchers had access to it.

### Data analysis

The recordings from the audio recorder were transcribed verbatim, and line-by-line analysis was conducted by reading the transcripts line-by-line as part of open coding. Line-by-line analysis ensured that the analysis is grounded on the findings, not on existing theoretical formulations (Corbin & Strauss 2015:105). The researchers revisited the emerging categories, identifying similarities and differences between and amongst them (Corbin & Strauss 2015:112; Tie et al. 2019:12). The next phase was axial coding, through which categories’ reduction took place. Emerging categories were organised into categories and subcategories according to the six elements (cause, content, contingencies, consequences, covariances and conditions) in the Corbin and Strauss data analysis model as depicted in Table 1 (Corbin & Strauss 2015:147).

**TABLE 1:** Themes and subthemes on how to improve simulation-based education in Lesotho.

Theme	Subtheme
Resources to support simulation-based education	**Human resources** Increasing number of clinical supervisors Engaging preceptors in simulation **Financial resources** Budgeting for equipment and supplies Increase number of manikins Procure good-quality manikins **Infrastructure** Increase number and sizes of the simulation laboratories
Teaching and learning process	**Educators’ duties and responsibilities** Conducting operational studies related to simulation Treating students with respect and avoid providing nonconstructive negative comments to students Being innovative and creative Ensuring realism in the simulation laboratory **Students’ desires** Being fully engaged in simulation preparation **Simulation time** Allocation of more time for simulation and open access to the simulation laboratory

### Trustworthiness

Lincoln and Guba trustworthiness criteria ensured scientific rigour of the study (Streubert & Carpenter 2011:60). To ensure credibility, the researchers spent a considerable amount of time with the participants during the interviews in order to build rapport, enabling them to voice their perceptions freely. Dependability was ensured by reporting all the processes within the study in detail, whilst confirmability was ensured by availing tape-recorded information of raw data, field notes and research report for an external audit. To ensure transferability, a thick description of the phenomena under investigation was performed by providing a detailed description on the number of participants in the field work, data collection methods that were employed, length of data collection sessions and period over which data were collected (Streubert & Carpenter 2011:60).

## Results

A total of 52 participants took part in this study. Educators were aged between 29 and 62 years. A majority of the educators 78.6% (*n* = 22) were female whilst 21.4% (*n* = 6) were male. Their nursing education experience ranged from 2 to 33 years. Students’ ages ranged between 18 and 40 years, and the majority of them 66.7% (*n* = 16) were female whilst few of them 33.3% (*n* = 8) were male. Each class (second-year, third-year and midwifery) was represented by two students. Data analysis revealed two categories: (1) resources to support SBE (human, financial and infrastructure) and (2) teaching and learning process (educators’ duties and responsibilities, students’ desires and simulation time). Table 1 summarises the categories and subcategories of the data.

### Category 1: Resources to support simulation-based education

In this study, participants showed that resources play a major role in ensuring quality simulation. The study revealed the following perceptions on resources: human resources, financial resources and infrastructure.

#### Human resources

The study participants explained that for simulations to run effectively, the NEIs need to increase the number of clinical supervisors and engage preceptors in SBE. Simulation-based education in Lesotho is led by nurse educators known as ‘clinical supervisors’ (NEPI 2012:12). These are individuals who conduct clinical teaching in the simulation laboratories and in the hospitals or clinics where nursing and midwifery students are placed. This is what some participants had to say in relation to increasing the number of clinical supervisors:

‘The number of clinical supervisors need to be increased from one per level to at least two per level.’ (Participant 2, nurse educator, FGD 1)

‘… I am also thinking of increased human resources, like the clinical supervisors as that number is limited to one per level.’ (Participant 10, nurse educator, FGD 2)

There was a consensus from the participants that preceptors need to be engaged in simulation. These are individuals who are practicing as nurses or midwives who also mentor students when placed in the clinical area. Here are some of the verbatim comments from the participants:

‘I think it is a very good idea to incorporate preceptors into simulation structure because the preceptors are the ones who are with students most of the time in the clinical areas.’ (Participant 15, nurse educator, FGD 3)

‘My addition is about increased engagement of preceptors to assist in student mentoring.’ (Participant 24, nurse educator, FGD 4)

#### Financial resources

In order to improve simulation experience for students, most participants explained that financial resources need to be taken into consideration. These include purchasing equipment and supplies, increasing number of manikins, purchasing quality manikins and procuring the latest high-fidelity manikins. Simulation-based education requires the use of real medical equipment such as sphygmomanometers and medical supplies such as cotton swabs, syringes and needles. In order to ensure that students get used to real medical equipment used in hospitals, NEIs need to procure such medical equipment. This is what some participants had to say in relation to purchasing equipment and supplies:

‘Some equipment that is being used such as the gloves, plastic aprons and other supplies are bought by the institution and those things are a little bit expensive.’ (Participant 18, nurse educator, FGD 3)

‘Sometimes we have to order medical supplies we use in the skills laboratory in South Africa as local suppliers have a tendency of not having the required supplies and that has an implication in the prices.’ (Participant 21, nurse educator, FGD 4)

Low, medium and high-fidelity manikins are used in SBE. Some participants voiced that in order to improve SBE, there is a need for the institutions to purchase more manikins which are of good quality with the latest technology. This will ensure that many students get chance to practice various competencies in time. This is what some participants had to say:

‘Let us say we are doing intramuscular injection and we are a class of 30, it’s better for us to have three or four manikins so that we divide ourselves.’ (Participant 28, nursing student, FGD 5)

‘The intramuscular injection manikin that I’ve talked about if they can improve it so that if it is pricked it doesn’t show that it has been pricked so that the students do not cram that they are going to give the injection there …’ (Participant 11, nurse educator, FGD 2)

#### Infrastructure

Some participants indicated that improving simulation infrastructure will ultimately improve students’ simulation experience. Participants explained that increasing the number of simulation rooms and the sizes of the simulation rooms will lead to improvement of SBE. A major setback in simulation was the size of the simulation rooms. Most participants explained that the space in the simulation room is limited and does not cater for a large number of students. This is what some participants had to say:

‘If funds allow, I could advocate for an expanded infrastructure.’ (Participant 001, PNE, in-depth face-to-face interview)

‘So, the issue of being congested in a small room in the simulation is the one that prevents practising altogether. But if the simulation gets extended, I guess we will be able to be in smaller groups.’ (Participant 40, midwifery student, FDG 7)

### Category 2: Teaching and learning process

This theme emerged as the participants explained that educators involved in SBE have a part to play in ensuring that SBE is improved. In addition, some participants also believed that students also have a part in improving SBE. This theme is discussed under the following headings: educators’ duties and responsibilities, students’ desires and time allocated for simulation.

#### Educator’s duties and responsibilities

Participants explained that for SBE to be successful, educators need to fulfil certain responsibilities. These responsibilities can be carried out on an individual basis or via teamwork. These responsibilities include conducting operational studies related to simulation, treating students with respect and avoiding negative comments to students, being innovative and creative and ensuring realism in the simulation laboratory. This is what some participants said in relation to conducting operational studies:

‘The other improvement that needs to be there is for us to conduct mini-operational studies to always find out where we need to improve in our simulation education … To get a feedback from the students and the users of simulation as to how do they feature …’ (Participant 2, nurse educator, FGD 1)

‘I think the educators need to provide us with surveys to assess if we are satisfied with their teaching in the simulation laboratory.’ (Participant 38, nursing student, FGD 7)

Some students expressed concern with how the nurse educators or clinical supervisors provide comments to them during simulation. They explain that some comments are negative and are belittling them. The students explained that some comments are unnecessary, and to ensure that they have best simulation experience, simulation facilitators need to provide positive feedback, not negative comments. This is what some students had to say:

‘Well, there are those comments that really frustrate you when you are performing a procedure like that one of saying “what are you doing …”’ (Participant 31, nursing student, FGD 6)

‘Some comments are not necessary, they are meant to belittle us, and it affects us, it really affects us.’ (Participant 25, nursing student, FGD 5)

Simulation needs a lot of creativity from the simulation facilitator. This will stimulate critical thinking and decision-making in student nurses. Some participants voiced that in order to improve SBE, there is a need for innovation and creativity. This is what some participants had to say:

‘There are those parts of the manikins that we can improvise with, like you know, use of sponges for suturing.’ (Participant 38, nursing student, FGD 7)

‘When artificially creating wounds to be dressed, it’s the matter of changing places, being innovative with the manikins and not just sticking on one procedure, it needs to be a bit more innovative.’ (Participant 32, midwifery student, FGD 6)

Some participants explained that simulation lacks realism. What happens in the simulation laboratory is very artificial and very different from what they see in real clinical practice. Verbatim comments from some of the participants are as follows:

‘I understand this is a simulation but sometimes it becomes so fake that it doesn’t make sense. I mean, if we are talking about amoxicillin, show me amoxicillin, not that I find tablet that I don’t even know the name.’ (Participant 36, nursing student, FGD 6)

‘The manikins are not performing like real people, so I like real patients.’ (Participant 44, nursing student, FGD 8)

#### Students’ desires

In order to fully implement and improve SBE according to the participants, the simulation facilitators need to consider students’ desires. One of the things that students desire during simulation is to be involved in simulation preparation. Some participants expressed a concern that they are not involved in simulation preparation. Most of the time during simulation, equipment, medical supplies and manikins are prepared by facilitators prior to simulation. This is what some participants said in relation to simulation engagement:

‘[*D*]uring practice, I think it’s our responsibility to take part in preparing equipment for the procedures that we’ll be doing, because sometimes we may find that whoever who has prepared for us, things are not well prepared or are not prepared in a way that we would have prepared them ourself.’ (Participant 26, nursing student, FGD 5)

‘I would like to be given a chance to set my own scenario and also attempt that scenario …’ (Participant 33, midwifery student, FGD 6)

#### Simulation time

Simulation time seems to be of importance to the students and educators. Study participants explained that in order to improve SBE, simulation needs to be allocated more time within the classroom timetables, as currently simulation is allocated between 2 h and 8 h in a week, depending on the institution. Students must also have open access to the simulation laboratory. An extract of direct verbatim comments from the participants are as follows:

‘We are sometimes given the keys to the simulation laboratory and we come after hours and during the weekends to practice.’ (Participant 30, nursing student, FGD 5)

‘I also think the rooms should always be opened for any student who wants to use the skills laboratory … like anytime, even during lunch time.’ (Participant 44, nursing student, FGD 8)

## Discussion

The aim of this study was to describe nurse educators’ and students’ perspectives on ways to improve implementation of SBE in Lesotho. The results revealed that in order to improve SBE implementation, resources that support simulation must be availed to the educators. In addition, teaching and learning process need to be conducted in such a manner that students are respected and educators know their roles as facilitators of learning.

### Resources to support simulation-based education

In this study, resources that support SBE included human resources, financial resources and infrastructure.

#### Human resources

In order to ensure a smooth simulation experience for the students, the study participants explained that the NEIs need to increase the number of clinical supervisors and engage preceptors to mentor students. According to Riabtseva, Reynolds and Gisin (2015:22), simulation laboratories need to be manned by a team of professionals. The advantage of manning up the simulation with two or more professionals is to reduce the risk of making poor decisions and assist in avoiding routine blindness. The team should be composed of clinical supervisors and educators who closely and regularly engage in clinical practice in order to guarantee that training sessions are realistic and dynamic. Published literature supports engagement of preceptors in SBE because preceptors can mentor students and assist in rotation of students during objective structured clinical examination (OSCE) in a timely manner (Obizoba 2018:81). According to Hill and Williams (2017:382), a preceptor is a qualified nurse who is given the responsibility to mentor one or two nursing students at a time.

#### Financial resources

Study participants explained that financial resources need to be taken into consideration in order for simulation to be successful and effective. These include budgeting for equipment and supplies, increasing number of manikins, purchasing quality manikins and procuring the latest high-fidelity manikins. A study conducted by Krishnan et al. (2017:88) found that during simulation, real medical equipment and supplies are used and they need to be budgeted for. According to Martins et al. (2018) and Larue et al. (2015:134), procuring high-fidelity manikins is costly for low-resource settings, and low-fidelity manikins can be opted for because they are cheaper. Lapkin and Lavett-Jones (2011:17) explained that use of high-fidelity simulators does not only present a financial challenge to low-resource settings, as even in Australia in 2010, only 45% of universities used high-fidelity simulation. Institutions who are resource limited can opt for low-fidelity simulation but pitch simulation scenarios to a level that stimulates critical thinking, problem-solving and decision-making.

#### Infrastructure

In this study, participants explained that there is a need to improve simulation infrastructure, as it has a direct influence on students’ learning. Simulation rooms must be increased in number and size. According to Guinez-Molinos et al. (2017:199), some simulation activities require the simulation facilitator to divide students into groups and then allocate them to various rooms. If there are not enough rooms, some planned activities may fail, hence it is important for the NEIs to improve simulation infrastructure. The simulation infrastructure must have various simulation rooms to accommodate manikins and 10–20 students at a time, and the size of each room can be 300–450 sf^2^ with storeroom, bathrooms, control office and audio-visual infrastructure (Baily 2020:14; Dodson, Adam & Stone 2017:33; Ross 2012:27). The simulation rooms will be used by the facilitators when they demonstrate patient care on the mannikins, students will practice and do return demonstrations in the simulation rooms and assessment will be also carried out in those simulation rooms. A storeroom will be used to keep medical and surgical supplies and task trainers. The control office will be used by the simulation facilitator to control some of the high-fidelity mannikins whilst watching students on the monitor when they are performing procedures. As for the audio-visual infrastructure, each simulation room must be fitted with cameras that can capture audio and video, and this will be used for teaching and learning and also for security purposes (Ross 2012:27).

### Teaching and learning process

To ensure success of SBE, educators and students have a role to play. Educators must professionally and properly exercise their responsibilities during simulation, students’ desires must be met and time allocation for simulation must be reconsidered.

#### Educator’s duties and responsibilities

Study results show that educators need to conduct operational studies related to simulation, treat students with respect, avoid providing nonconstructive comments to students, be innovative and creative and ensure realism in the simulation laboratory. Conducting research as one of the responsibilities of the educator may have an impact on the quality of teaching. According to Médecins Sans Frontières (2013:8), operational research is important because it can improve teaching and learning as gaps are identified. One of the responsibilities of the educators is to treat students with respect and provide constructive feedback. Some nursing students experience anxiety during SBE and need support (Landeen et al. 2015:488). In other students, the anxiety is caused by the fact that they are being watched. If the students are already anxious and the facilitators provide non-constructive comments, this can have a negative impact on student learning (Baptista et al. 2016:16). Debriefing needs to be conducted in a nonjudgemental manner; the facilitator must discuss positive aspects observed during simulation and areas that need improvement (Martins et al. 2018:9).

Simulation needs a lot of creativity from the simulation facilitators, as explained by some of the participants. According to Krishnan et al. (2017:85), there are various ways in which the simulation facilitator can show innovativeness, and this includes utilisation of various teaching methodologies during simulation. The innovation starts from creating simulation scenarios that resemble real life. Park et al. (2013:45) also explained that authentic cases need to be developed by the simulation facilitator, and that is where innovations come in. Some participants explained that the simulations lack realism and what happens in the simulation laboratory is very artificial and very different from what they see in real clinical practice. It is not only in Lesotho where students are saying that simulation lack realism. Studies conducted by Krishnan et al. (2017:85), Au et al. (2016:20) and Larue et al. (2015:135) explained that students have negative attitudes towards simulation because of lack of realism. This is because students see simulation as artificial and it is not close to the real clinical environment.

#### Students’ desires

During simulation, students’ desires need to be considered. One of the things that students desire during simulation is to be actively involved in simulation preparation. In simulation, adult learners are mentored to become professionals. Merriam and Bierema (2014:52) explained that adult learners are self-directed and are actively engaged in their learning. Guinez-Molinos et al. (2017:197) explained that students can be actively involved in simulation preparation by utilising the collaborative clinical simulation approach. This is where small groups are utilised to collaboratively design and perform activities in simulated environments. Active involvement of students in simulation leads to reduced anxiety and increased confidence and enhances therapeutic communication (Carpenter et al. 2018:238; Palmer & Ham 2017:282).

#### Simulation time

The study results show that simulation time needs to be increased in order to ensure full utilisation of the simulation laboratories and improve students’ experiences. The participants explained that simulation time can be increased by ensuring open access to the simulation laboratory. This is where students can have access to the simulation laboratory at any time they wish to practice, as they are adult learners who are self-directed. Aebersold (2018:7) explained that in order to ensure full utilisation of simulation, simulation time can be increased by integrating simulation as a standalone course or module with its own assessment.

#### Limitations

Only the four private NEIs were included in this study, and the results may not be generalised. First-years were not included in the study as the institutions did not have new students’ intake because of COVID-19 restrictions.

## Conclusion

This study highlighted gaps such as a limited number of clinical facilitators, nonconstructive feedback from the educators and little time allocated for simulation, and those gaps need to be addressed in order to effectively implement SBE. Participants acknowledged that there are limited numbers of simulation facilitators. Increasing the number of simulation facilitators will provide students with a chance of continued mentoring in the simulation laboratory. Students are very concerned about the comments of their educators during simulation, as some of the comments are belittling them. Proper debriefing after simulation needs to be carried out and educators need to treat students with respect. Lastly, participants (students) expressed concern that the time scheduled for simulation is limited. Nursing Education Institutions need to explore ways in which students can have open access to the simulation laboratory so that they can practice on their own in their spare time, as they are adult learners.
